# The Structure–Activity Relationship and Anticoagulation Mechanism of Polyglycerol Sulfates of Different Architectures

**DOI:** 10.1021/acs.biomac.6c00308

**Published:** 2026-04-29

**Authors:** Clemens Krage, Marie Weinhart, Benjamin F. L. Lai, Anja Stöshel, Katharina Achazi, Jayachandran N. Kizhakkedathu, Rainer Haag

**Affiliations:** † 9166 Institut für Chemie und Biochemie, Freie Universität Berlin, D-14195 Berlin, Germany; ‡ Institute of Physical Chemistry and Electrochemistry, 26555 Leibniz Universität Hannover, Hannover 30167, Germany; § Centre for Blood Research, Life Sciences Institute, Department of Pathology and Laboratory Medicine, 8166 University of British Columbia, 2350 Health Sciences Mall, Life Sciences Centre, Vancouver, BC V6T 1Z3, Canada; ∥ The School of Biomedical Engineering, University of British Columbia, 2350 Health Sciences Mall, Life Sciences Centre, Vancouver, BC V6T 1Z3, Canada

## Abstract

Although unfractionated heparin (UFH) remains a vital agent for rapid anticoagulation, its reliance on animal-derived sources results in batch-to-batch variability and contamination risks, and its clinical application is further restricted by the possibility of heparin-induced thrombocytopenia. Dendritic polyglycerol sulfates were investigated as heparin analogues in 2004 due to their ability to mimic its charge, which is essential for its acting mechanism, and revealed an anticoagulant activity of 15–35% compared to UFH. In the current study, we found that the anticoagulant effect of polyglycerol sulfates further increases with their flexibility, resulting in a comparable activity of linear polyglycerol sulfate to UFH. Furthermore, we comprehensively analyzed the mechanism of action and discovered an antithrombin-independent, thrombin-selective mechanism. Moreover, we confirmed that FDA-approved protamine sulfate is a viable reversal agent for polyglycerol sulfate.

## Graphical abstract

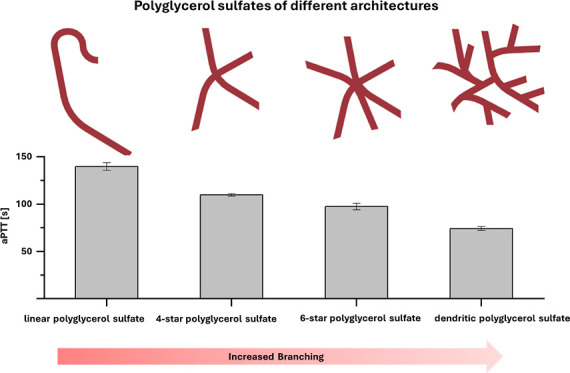

## Introduction

Anticoagulants play a crucial role in the prevention of thrombotic events, for example, in acute coronary syndrome (ACS) and the stabilization of deep vein thrombosis (DVT). The anticoagulant activity of heparin was discovered in the early 1900s and heparin plays a critical role in anticoagulant therapy to this day.^1–4^ Heparins are highly negatively charged glycosaminoglycans composed of alternating units of N- and O-sulfated glucosamine and glucuronic or iduronic acid. Unfractioned Heparins (UFHs) have an average molecular weight of around 16 kDa and are currently mostly purified from porcine intestinal mucosa.^5,6^ Other types of heparins used in the clinics include low molecular weight heparins (LMWHs) (3 kDa to 7 kDa) and the synthetic pentasaccharide fondaparinux.^7^

Heparin acts as an anticoagulant by interacting with the coagulation cascade via two separate mechanisms: one allosteric and one bridging mechanism. In the bridging mechanism, heparin forms a ternary complex with antithrombin III (ATIII) and thrombin (FIIa) by bridging both proteins via electrostatic interactions. In the allosteric mechanism, heparin binds to ATIII, leading to a conformational change in the active site, which then binds to factor Xa (FXa). Both pathways lead to a reduced FIIa-mediated fibrin activation, which is responsible for the blood clot formation.^8^ While both pathways can be activated by UFH, LMWHs are less apt to perform the bridging mechanism necessary for ATIII-FIIa binding due to their reduced chain length and are thus more FXa-specific.

LMWHs such as enoxaparin, tinzaparin, and dalteparin are generated by enzymatic cleavage of UFH leading to a reduced molecular weight range from 4.5 to 6.5 kDa.^9^ The possibility of subcutaneous (s.c.) application and a longer half-life of LMWH (enoxaparin, tinzaparin, dalteparin) over UFH make LMWH preferably useful for long-term prophylaxis of thrombotic events. Further optimizations of LMWH led to the development of the synthetic pentasaccharide fondaparinux consisting of the minimum chain length for ATIII-binding.^10^ While this specific pentasaccharide reduces the variation in batch activity, the complex manufacturing process of this fully synthetic molecule makes it cost-intensive.^11^

In contrast to the s.c. applicable LMWHs, UFH requires intravenous administration and is favored when rapid onset, short duration, and full reversibility are needed, for example, during surgical procedures to reduce the bleeding risk. UFH activity can be reversed by the injection of protamine, a highly basic polypeptide that binds to heparin via electrostatic interactions but requires a certain chain length of heparin, leading to reduced reversal of LMWH or fondaparinux.^12–16^

A further drawback of the long-term application of UFH is a high risk of type II heparin-induced-thrombocytopenia (HITII) development.^17^ The mechanism behind HITII is the binding of heparin to platelet-factor 4 (PF4), exposing an immunogenic epitope and triggering the formation of antibodies to this complex.^18,19^ This trimeric complex, in turn, activates and depletes platelets, leading to a paradox hypercoagulative state with increased risk of thrombosis.

These drawbacks, alongside with the problematic batch-to-batch heterogeneity and possibility of pathogen contamination due to their animal origin, led to the development of further anticoagulant compounds.^11^ The vitamin K antagonists phenprocoumon and warfarin are a valuable alternative but require a tedious surveillance of blood clotting time and diet restrictions and have no quick-acting reversal agent. This led to the recent development of the direct oral anticoagulants (DOACs) rivaroxaban, apixaban (anti Xa), and dabigatran (anti IIa), which were milestones in anticoagulant therapy with correspondent reversal agents (andexanet (removed recently from the market) and idarucizumab) due to their more rapid onset, a wider therapeutic window, and less dietary and drug restrictions.^6^

Despite these breakthroughs in anticoagulant therapy, heparins are still highly clinically relevant when a rapid onset and short half-life is desired due to acute illnesses, surgery, or in cases of renal insufficiency.^6,20^ Thus, there remains a need for alternatives to heparins that are less susceptible to HITII and less prone to batch-to-batch variability and contamination risk resulting from animal origin. One successful experimental approach was the recently published reversible peptide nucleic acid-linked direct thrombin inhibitors as UFH alternatives.^21^

In 2004, Türk et al. discovered that highly sulfated dendritic polyglycerol (dPGS) can mimic the negative charges of heparin and has an anticoagulant activity of 15–35% compared to UFH in platelet poor plasma (PPP).^22^ Polyglycerol (PG) and its sulfated derivatives (PGS) exhibit high bio- and hemocompatibility, and PG is structurally related to the FDA-approved polyether polyethylene glycol (PEG).^23,24^ The additional hydroxyl group per monomer, however, enables a functionalization of the polymer for multivalent interactions, a concept that has been employed for the targeting of a range of target proteins.^23–29^ The biocompatibility of PG is further demonstrated by the FDA’s regulatory approval of PG oligomers, including up to decamers, for use as food and pharmaceutical additives.^30^

Based on the previous discovery of the anticoagulant activity of dPGS, we aimed to investigate the effect of increased polymer flexibility by switching from the dendritic structure to partially branched polyglycerol sulfate analogues or linear polyglycerol sulfate (lPGS) to better mimic the linear structure of heparins. This allowed us to observe a positive association between chain flexibility and anticoagulant activity, with lPGS displaying the strongest effect at a potency similar to UFH. We then analyzed the structure–activity relationship in detail to determine the interaction mechanisms of lPGS with ATIII, FIIa, and FXa.

## Materials and Methods

### Synthesis

The synthesis of dendritic and linear PG has been extensively described previously.^24,27,31–33^ Four-arm star PG and six-arm star PG were synthesized by adjusting the initiator molecule of the linear polyglycerol synthesis to pentaerythritol and dipentaerythritol. The sulfation was achieved with a pyridine-sulfur trioxide complex, and the functionalization degree was determined by elemental analysis. All polymers were purified by dialysis. The detailed synthesis of the polymers is described in the Supporting information (Scheme S1–S3).

### Activated Partial Thromboplastin Time (aPTT)

Blood samples were collected in citrated vacutainer tubes from BD (9:1 v/v blood to buffered sodium citrate solution). The blood collection protocol was approved by the UBC clinical ethical board (H20–00084) and all participants gave informed consent. The samples were centrifuged (20 min, 1200*g*) at room temperature. The platelet-poor-plasma (PPP) was then separated and used immediately for the aPTT assay with a ST4 automated hemostasis coagulation analyzer (Diagnostic Stago, Inc.) with mechanical end point determination. PPP (180 μL) was mixed with PGS (20 μL) in PBS at varying concentrations. Then, partial thromboplastin reagent (actin FSL from Dade Behring) was added at a 1:1 ratio. 100 μL of this mixture was warmed to 37 °C. Subsequently, 50 μL of prewarmed 0.025 mM calcium chloride solution was added, and the time was recorded until a fibrin clot was formed at 37 °C. The PGS were compared with UFH, tinzaparin, and a PBS control. The experiments were terminated after 500 s. Interexperimental deviations of the aPTT are a result of the performance of these studies in different laboratories with their respective instruments and different batches of the analyzed polymers.

### Thromboelastography (TEG) Measurements

TEG experiments were measured on a thrombosis viscoelastic analysis system (ImproveClot T-400, Improve Medical Instruments Co., Ltd., China). Citrated whole blood (400 μL) was added to the samples dissolved in PBS, recalcified by the addition of a CaCl_2_ solution (23.5 μL, final concentration: 11 mM), and transferred into the TEG cup.

#### Cytotoxicity Assay

Cytotoxicity was investigated for human umbilical vein endothelial cells (HUVEC) (ThermoFisher) via an MTS assay. 10,000 HUVEC cells per well were cultured in EGM-2 medium (ThermoFisher) in the presence of PGS for 48 h at 37 °C. Then, toxicity was analyzed with an MTS assay kit (Abcam) according to the manufacturer’s instructions.

#### Chromogenic Assays

The activity of FXa and FIIa in the presence of anticoagulants was determined by chromogenic assays purchased from Abcam and performed according to the manufacturer’s instructions. Briefly, a chromogenic substrate of the respective protein was added, leading to the development of a dye detectable by a UV–vis plate reader. In the case of allosteric or competitive binding of, e.g., lPGS, the rate of development of detectable dye was reduced.

#### Surface Plasmon Resonance (SPR) Studies

All experiments were performed on a Biacore X100 (GE Healthcare) with standard HBS-EP (150 mM NaCl, 10 mM HEPES, 3 mM EDTA, 0,05% P20, pH 7.4) as a running buffer. For the ATIII immobilization, ATIII (15 μg/mL, Merck) was dissolved in a 10 mM sodium acetate buffer at pH 5.2 and injected over a previously EDC/NHS-activated CM5 Biosensor (GE Healthcare) until 1000 RUs were reached by the standard protocol of the Biacore control software. For FIIa immobilization, biotinylated FIIa (200 nM, ThermoFisher) was injected over an SA Biosensor (GE Healthcare) until 700 RUs were reached. For protamine immobilization, protamine (50 μg/mL, Merck) was dissolved in a 10 mM sodium acetate buffer (pH, 4.5) and injected over a previously EDC/NHS-activated CM5 Biosensor (GE Healthcare) for 1080 s by the standard protocol of the Biacore control software.

The binding of UFH, lPGS, and dPGS was investigated by sample injection for an association time of 120 (ATIII) or 180 s (FIIa, protamine) and a dissociation time of 600 (ATIII, protamine) or 300 s (FIIa) followed by a 30 s pulse of regeneration solution (2 M NaCl). For FIIa and ATIII, the equilibrium dissociation constant K_D_ was determined by “affinity fitting” with the BIAevaluation software. Here, the response level was plotted against the RU at the steady state and the K_D_ was defined as the c where RU = *R*_max_/2, assuming a Langmuir adsorption isotherm. For protamine, the equilibrium dissociation constant K_D_ as well as the association (*k*_a_) and dissociation constant (*k*_d_) were determined by “kinetic fitting” with the BIAevaluation software using a 1:1 binding model. This approach was chosen for the investigation of the protamine binding because no steady state was reached during the association time, making affinity fitting impossible.

## Results and Discussion

For the investigation of the effect of the architecture of the PGS on the anticoagulant activity, we synthesized variations of the PGS with similar molecular weight backbones (5 kDa) while varying between the linear, dendritic, and intermediate 4-arm star or 6-arm star-shaped scaffolds (Figure 1 A–D, Table 1). The synthesis of lPGS and dPGS is extensively described in previous literature.^24,31^ The architecture of the star-shaped PG was realized by adjusting the initiator molecule for linear PG to pentaerythritol for four-arm star PG and dipentaerythritol for six-arm star PG. The detailed synthesis is described in Figures S1–S10.

**Figure 1:**
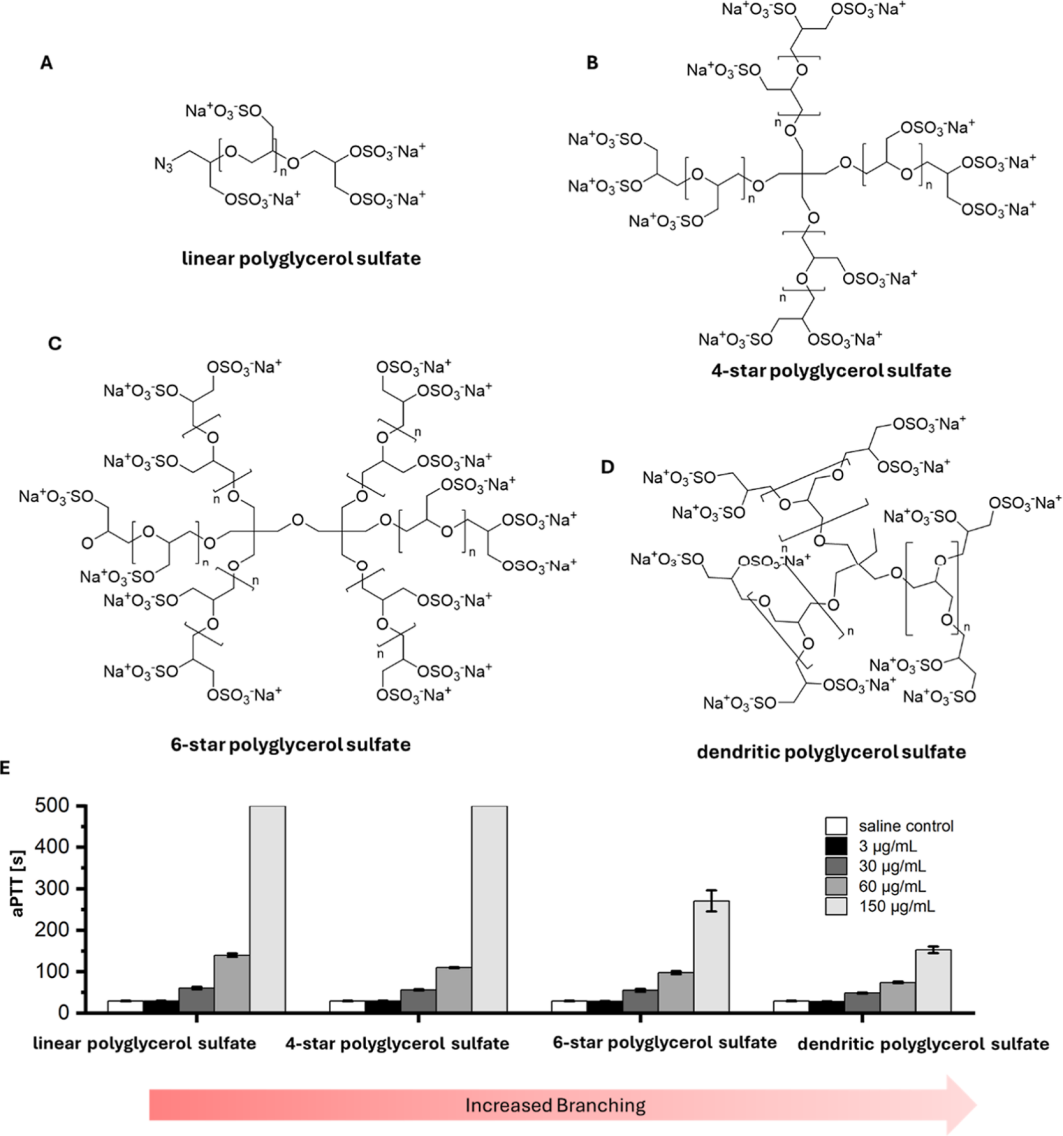
Architecture of linear (A), 4-star (B), 6-star (C) and dendritic (D) polyglycerol sulfate. (E) Activated partial thromboplastin time (aPTT) of platelet poor plasma. Measurements were terminated after 500 s. All experiments were performed as 3 technical replicates each, thus total 9 data points. All data represent mean ± standard deviation.

**Table 1:** Synthesized Polyglycerol Sulfates of Different Length, Architecture, and Degree of Sulfation

sample	scaffold	core *M*_n_ (kDa)^a^	PDI^b^	sample *M*_n_ (kDa)^c^	DS (%)^d^ -	sulfate groups^e^
lPG_2 kDa_S_17_	linear	1.9	1.15	3.6	63	17
lPG_3 kDa_S_44_	linear	3.2	1.09	7.7	100	44
lPG_5 kDa_S_19_	linear	5.0	1.07	7.2	28	19
lPG_5 kDa_S_36_	linear	5.0	1.07	9.3	54	36
lPG_5 kDa_S_60_	linear	5.0	1.07	12.1	90	60
lPG_10 kDa_S_135_	Linear	10	1.09	25.9	100	135
lPG_25 kDa_S_94_	Linear	25	1.11	36.1	28	94
lPG_25 kDa_S_162_	Linear	25	1.11	44.1	48	162
lPG_25 kDa_S_337_	Linear	25	1.11	64.8	100	337
lPG_54 kDa_S_625_	Linear	54	1.31	118	85	625
dPG_5 kDa_S_65_	hyberbranched	5.0	1.60	9.6	90	65
4-star PG_5 kDa_S_67_	Branched	5.0	1.09	11.8	100	67
6-star PG_5 kDa_S_67_	Branched	5.0	1.05	11.8	100	67

a Median molecular weight (*M*_n_) of the unfunctionalized PG determined by gel permeation chromatography (GPC).

b Polydispersity index (PDI) of the unfunctionalized PG determined by GPC.

c *M*_n_ of the sulfated samples calculated with the DS.

d Degree of sulfation (DS) determined by elemental analysis.

e Amount of sulfate groups calculated from the DS.

The influence on the anticoagulant activity was investigated by determination of the aPTT in platelet-poor-plasma (PPP), revealing a rise in activity with an increase of linear component within the polymers: while 60 μg/mL dPGS prolonged the aPTT to 74.2 s in comparison to control PPP at 30.1 s, increasing the linear components resulted in an increase of the aPTT of 97.4 s for 6-star PG_5 kDa_S_67_, 109.7 s for 4-star PG_5 kDa_S_67_, and 139.6 s for lPG_5 kDa_S_60_ (Figure 1E). This increase in anticoagulant activity is most likely attributable to the enhanced flexibility of the polymer, which may facilitate a greater number of interactions with its target molecule.

Following the observation that the linear architecture of lPGS exhibited the highest anticoagulant activity, we proceeded to determine the optimal polymer size. The synthesized dPGS of different sizes are presented in Table 1. Here, moderate anticoagulant effects were observed for lPGS_2 kDa_S_17_ starting at 25 μg/mL (Figure 2A). An increase in chain length led to a markedly increased anticoagulant effect, yielding a comparative aPTT of lPG_3 kDa_S_64_ to tinzaparin and of lPGS_5 kDa_S_60_ to UFH. The aPTT increased with the molecular weight up to lPG_10 kDa_S_135_, while a further increase in lPGS length had no effect on the aPTT. Additional investigations for the optimal degree of sulfation (DS) revealed the highest activity for the highly sulfated lPGS and negligible anticoagulant activity of linear PG with the lowest DS (28% DS) (Figure 2B). These findings emphasize the role of electrostatic interactions between sulfate groups and basic residues on the target proteins. They also align with previous strategies in which electrostatic interactions between polyanionic polymers and proteins bearing positively charged surface regions were exploited for targeting and increased binding was observed at higher functionalization degrees.^24,25,34^

**Figure 2:**
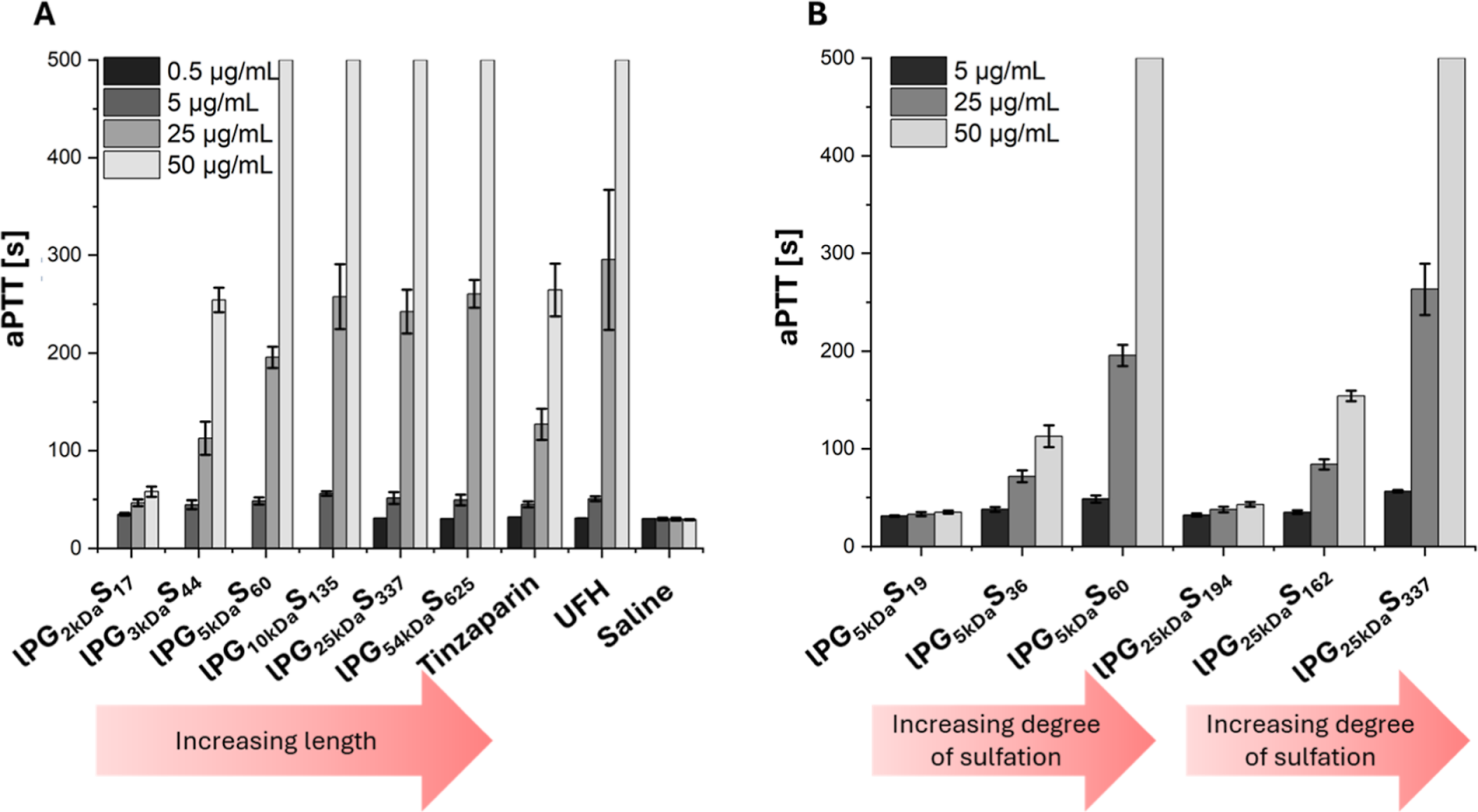
(A) Influence of lPGS chain length (0.5 μg/mL, 5 μg/mL, 25 μg/mL, 50 μg/mL) on blood clotting time in platelet-poor-plasma (PPP) by measurement of activated partial thromboplastin time (aPTT) compared to UFH and tinzaparin (0.5 μg/mL, 5 μg/mL, 25 μg/mL, 50 μg/mL). (B) Influence of degree of sulfation of lPGS (5 μg/mL, 25 μg/mL, 50 μg/mL) on clotting time of PPP. Measurements were terminated after 500 s. All experiments were performed as biological triplicates (*n* = 3 with 3 technical replicates each, thus total 9 data points). All data represent mean ± standard deviation.

To validate the observation of an increased anticoagulant effect with reduced polymer branching, TEG measurements were conducted in whole blood (Figure S11), as aPTT is performed in platelet-poor plasma (PPP) where activation is triggered by kaolin.

TEG, however, is performed in whole blood presenting a more physiological platform and allows for the quantification of further parameters (Table 2) like the initiation of clot formation (R time), time from the initiation to a clot size of 20 mm (K value), and clot strength (MA), while also allowing us to follow an endogenous fibrinolysis. Here, the previously observed effects in Figure 1 were confirmed for lPGS_5 kDa_S_60_ and UFH: while the control R-value of 9.4 min increased to 16 min following the addition of 0.05 mg/mL dPGS_5 kDa_S_65_, both lPGS_5 kDa_S_60_ and UFH completely inhibited coagulation for at least 2 h (Table 2).

**Table 2:** TEG Parameters

sample	C	*R* value [min]^a^	*K* value [min]^b^	MA [mm]^c^	α-angle^d^ -
PBS control	-	9.4 ± 2.7	2.7 ± 0.7	59 ± 4	54 ± 6
lPGS_5 kDa_S_60_	0.05 mg/mL	154 ± 27	32 ± 11	34 ± 4	5.7 ± 2.1
dPGS_5 kDa_S_65_	0.05 mg/mL	16 ± 3	2.8 ± 0.9	58 ± 8	53 ± 4
UFH	0.05 mg/mL	>120	-	-	-
LMWH	0.05 mg/mL	>120	-	-	-

a *R* value describes the initiation of clot formation.

b *K* value describes the time from initiation to a clot size of 20 mm.

c MA (maximum amplitude) describes the clot strength.

d α-angle is the angle between the baseline and the tangent to the steepest part of the rising curve.

To verify the cell compatibility, cytotoxicity experiments were conducted with HUVEC cells via an MTS assay and showed no cytotoxic effect within the concentrations relevant for anticoagulant therapy (Figure S12). Cytotoxic effects were only observed at concentrations above 2.5 mg/mL which is approximately 100× larger than an effective anticoagulant concentration, thus granting a rather large therapeutic window. Comparable data considering the cytotoxicity of PGS has been previously published.^24,27^

To gain mechanistic insights into the anticoagulant activity, we investigated whether the observed effect was mediated through the inhibition of FXa or FIIa in the presence of ATIII, using chromogenic assays (Figure 3A,B). These experiments demonstrated that PGS lacks interaction with FXa, while showing pronounced interaction with FIIa. Surprisingly, dPG_5 kDa_S_65_ interacted stronger with FIIa than lPG_5 kDa_S_60_, which is not in concordance with the aPTT and TEG experiments performed before. This suggests a higher availability of lPG_5 kDa_S_60_ compared to dPG_5 kDa_S_65_ in plasma and whole blood since the chromogenic assay is performed in pure buffer.

**Figure 3:**
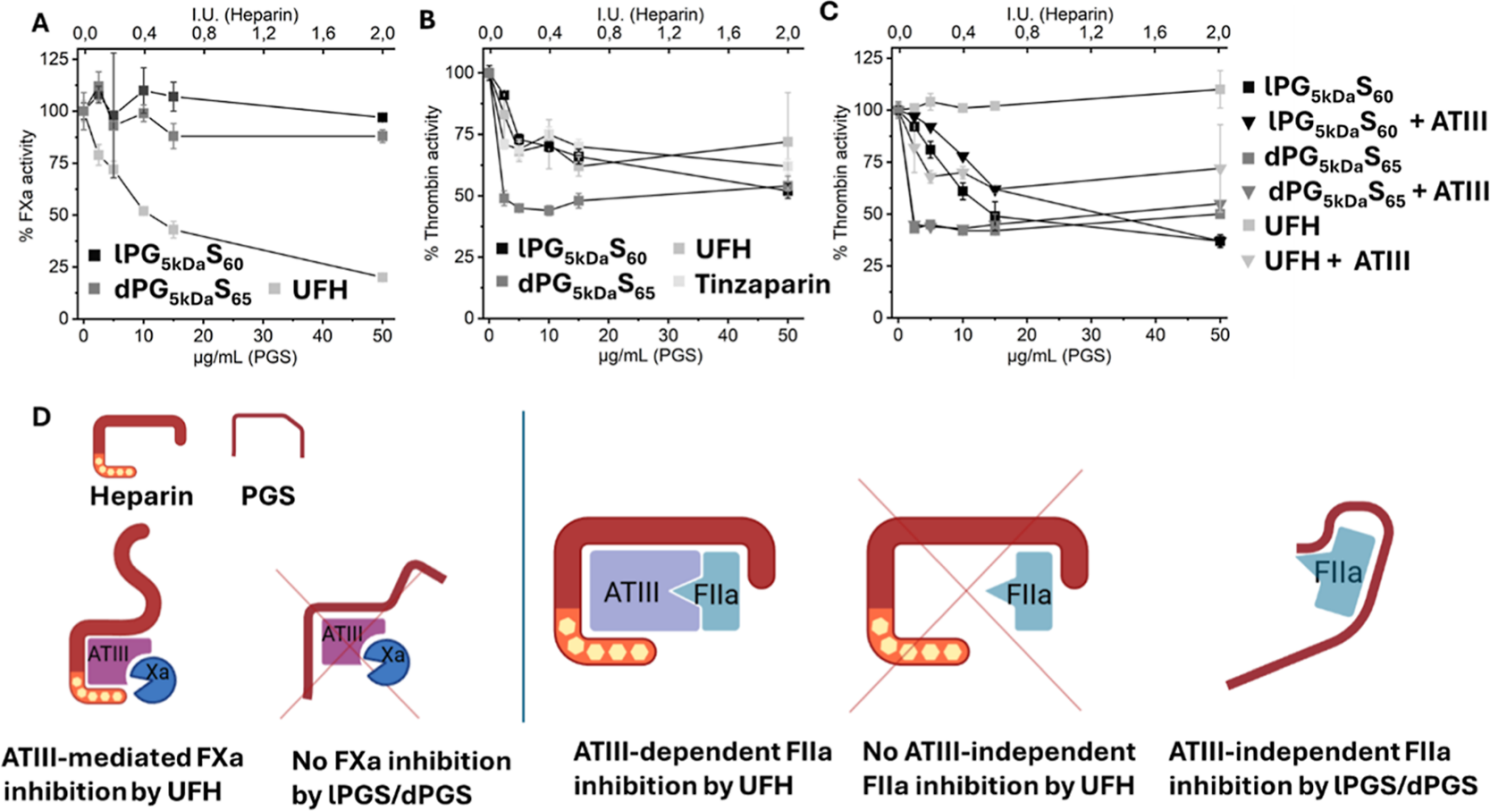
Investigation of the anticoagulation mechanism. (A) Chromogenic assay of FIIa activity in the presence of ATIII after adding dPG5_kDa_S_65_, lPG_5 kDa_S_10_, or UFH. (B) FXa activity in the presence of ATIII after adding dPG5_kDa_S_65_, lPG_5 kDa_S_10_, UFH, or tinzaparin. (C) FIIa activity in the presence and absence of ATIII after adding dPG5_kDa_S_65_, lPG_5 kDa_S_10_, or UFH. (D) A cartoon showing the potential anticoagulation mechanism: In contrast to UFH, PGS does not inhibit FXa activity. However, PGS inhibits FIIa activity in an ATIII-independent manner, while UFH only inhibits FIIa activity in the presence of ATIII. All data represent mean ± standard deviation, *n* = 3.

We initially attributed the stronger anticoagulant effect of lPGS compared to their dendritic counterparts in aPTT and TEG assays to their better fit with the linear heparin-binding site on the ATIII surface, leading to an ATIII-dependent effect. Additionally, their greater molecular flexibility was believed to facilitate bridging between ATIII and FIIa, similar to how UFH achieves its anticoagulant effect.^1^ Unexpectedly, the investigation of FIIa activity in the presence of lPG_5 kDa_S_60_ or dPG_5 kDa_S_65_, and the absence of ATIII (Figure 3C), also revealed a significant reduction in FIIa activity. This finding invalidates the hypothesis of an ATIII-dependent effect of PGS, as the addition of ATIII did not produce any further reduction in FIIa activity. Instead, it indicates a selective, direct (ATIII-independent) FIIa inhibition.

Direct, FIIa-selective inhibition has previously been reported for sulfated seaweed-derived β-l-arabinopyranose, whereas other sulfated polysaccharides, such as fucoidan, were shown to inhibit both FXa and FIIa.^35,36^ In addition, sulfated arabinogalactans have been reported to inhibit FIIa and FXa in an ATIII-dependent manner.^37^ These divergent findings among structurally distinct sulfated polymers underscore the critical role of the polymer scaffold in determining the underlying anticoagulant mechanism. Notably, despite sharing a high degree of sulfation, these materials exert their anticoagulant effects via different pathways. Other previous studies investigating sulfonated polymers with varying backbone architectures further indicate that the presence of a uronic acid backbone is a key determinant for enhanced ATIII activity.^38^ This observation is consistent with our findings, which demonstrate ATIII-independent anticoagulant activity in the absence of uronic acid structures.

To confirm the ATIII-independent effects observed in the chromogenic assays, we examined the interaction of the sulfated polymers with ATIII and FIIa using SPR spectroscopy (Table 3, Figures 4, S13, and S14). ATIII and FIIa were immobilized on the SPR sensors as described in the methods section. The binding analysis for ATIII supported the conclusions from the chromogenic assays: high-affinity binding was detected only for UFH (K_D_ = 18 nM), whereas lPG_5 kDa_S_60_ and dPG_5 kDa_S_65_ displayed only weak interactions in the millimolar range (K_D_ > 1 mM). This assumption is drawn from the absence of steady-state saturation as shown in Figure 4B,C, even at the highest tested concentration of 500 μM and 1000 μM. The affinity determined for UFH toward ATIII aligns well with previously reported values (18 nM vs 24–48.8 nM).^39,40^ These experiments were followed by the investigation of binding to FIIa, which demonstrated that all UFH, lPG_5 kDa_S_60_, and dPG_5 kDa_S_65_ bind to FIIa in the nanomolar range (K_D_ = 0.8–3.9 nM). The K_D_ determined for UFH binding to FIIa is approximately 1 order of magnitude lower than previously reported: 2.3 nM vs 61 nM.^41^ The most plausible explanation for this discrepancy is the variability between the particular heparin and FIIa batches employed. The higher affinity of lPG_5 kDa_S_60_ (K_D_ = 0.8 nM) relative to dPG5_kDa_S_65_ (K_D_ = 3.9 nM) is most likely attributable to its greater structural flexibility, which increases the effective interaction area compared to the more spherical dPG architecture. Its enhanced binding relative to that of UFH (2.8 nM) is likely driven by the combined effects of increased flexibility and higher charge density. Interestingly, the observation that PGS do not bind to ATIII highlights the effect of nonelectrostatic interactions of UFH with ATIII and the contribution of other binding mechanisms. This is in line with previous observations that only a subset of the basic residues along the 50 Å long positively charged patch on the ATIII surface is critical for heparin binding.^42^

**Table 3:** Affinity toward ATII and FIIa Determined by Surface Plasmon Resonance Spectroscopy (SPR)

K_D_ ^a^ [nM]	UFH	lPG_5 kDa_S_60_	lPG 1.70	dPG_5 kDa_S_65_	dPG -
ATIII	18	>10^6^ ^b^	n. b^c^	>10^6^ ^b^	n.b
FIIa	2.3	0.8	n. b	3.9	n.b

a *K*_D_ (equilibrium dissociation constant) was determined by SPR spectroscopy affinity fits using the BIAevaluation software.

b Affinity fit in the pseudolinear range of the Langmuir isotherm suggests a K_D_ in the millimolar range.

c No binding observed.

**Figure 4:**
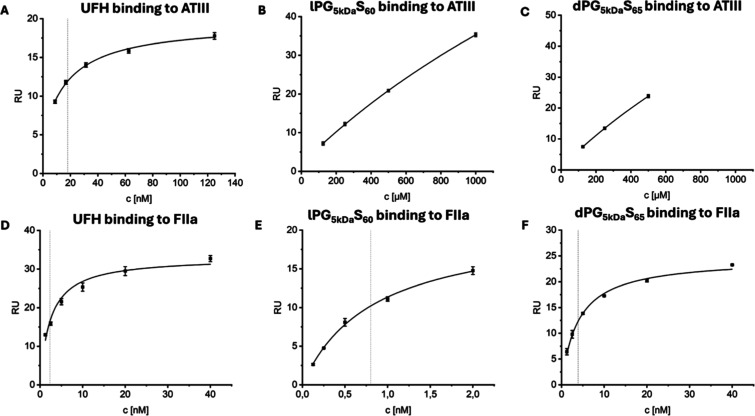
(A–C): SPR affinity fit sensorgrams determining the affinity of ATIII to (A) UFH (K_D_ = 18 nM), (B) dPG_5 kDa_S_65_ (K_D_ > 1 mM), and (C) lPG_5 kDa_S_60_ (K_D_ > 1 mM). (A–C) were performed as duplicate measurements. (D–F): SPR affinity fit sensorgrams determining the affinity of FIIa to D) UFH (K_D_ = 2.3 nM), (E) lPG_5 kDa_S_60_ K_D_ = (0.8 nM), and (F) dPG_5 kDa_S_65_ (K_D_ = 3.9 nM). All equilibrium dissociation constants are listed in Table 3. (D–F) were performed as triplicate measurements. The SPR raw data is shown in Figures S13 and S14. The dotted line represents K_D_ as determined by the affinity fit.

To determine the reversibility of these anticoagulant effects, we investigated the interaction of lPGS with protamine. Protamine is an FDA-approved heparin reversal agent with a highly positive surface potential and interacts electrostatically with the negatively charged heparin, suggesting a similar mechanism with PGS.^43^

The aPTT of PPP was investigated in the presence of lPGS_5 kDa_S_60_ (50 μg/mL) and the potential reversal agent protamine at varying ratios (2:1, 1:1, 1:2, 1:4) (Figure 5, Table 4). These studies confirmed the potential of protamine as a lPGS reversal agent: a 1:2 ratio of lPGS_5 kDa_S_60_ to protamine resulted in a reduction of the aPTT to 45.6 s compared to the aPTT of >500 s in the absence of protamine. The surprisingly large aPTT of >500 s at a 1:4 ratio of lPGS_5 kDa_S_60_ to protamine can be explained by the paradoxical anticoagulant effect that protamine itself has without a polyanion counterpart.^15,43,44^ The reversal effect was also confirmed by the TEG analysis of whole blood: Here, a 2.5:1 ratio of lPGS_5 kDa_S_60_ to protamine reduced the R-value of lPGS_5 kDa_S_60_ from 140.2 to 37.9 min, while a 2:1 ratio of UFH to protamine reduced the *R*-value to 87.7 min (Figure S15 and Table S1).

**Figure 5:**
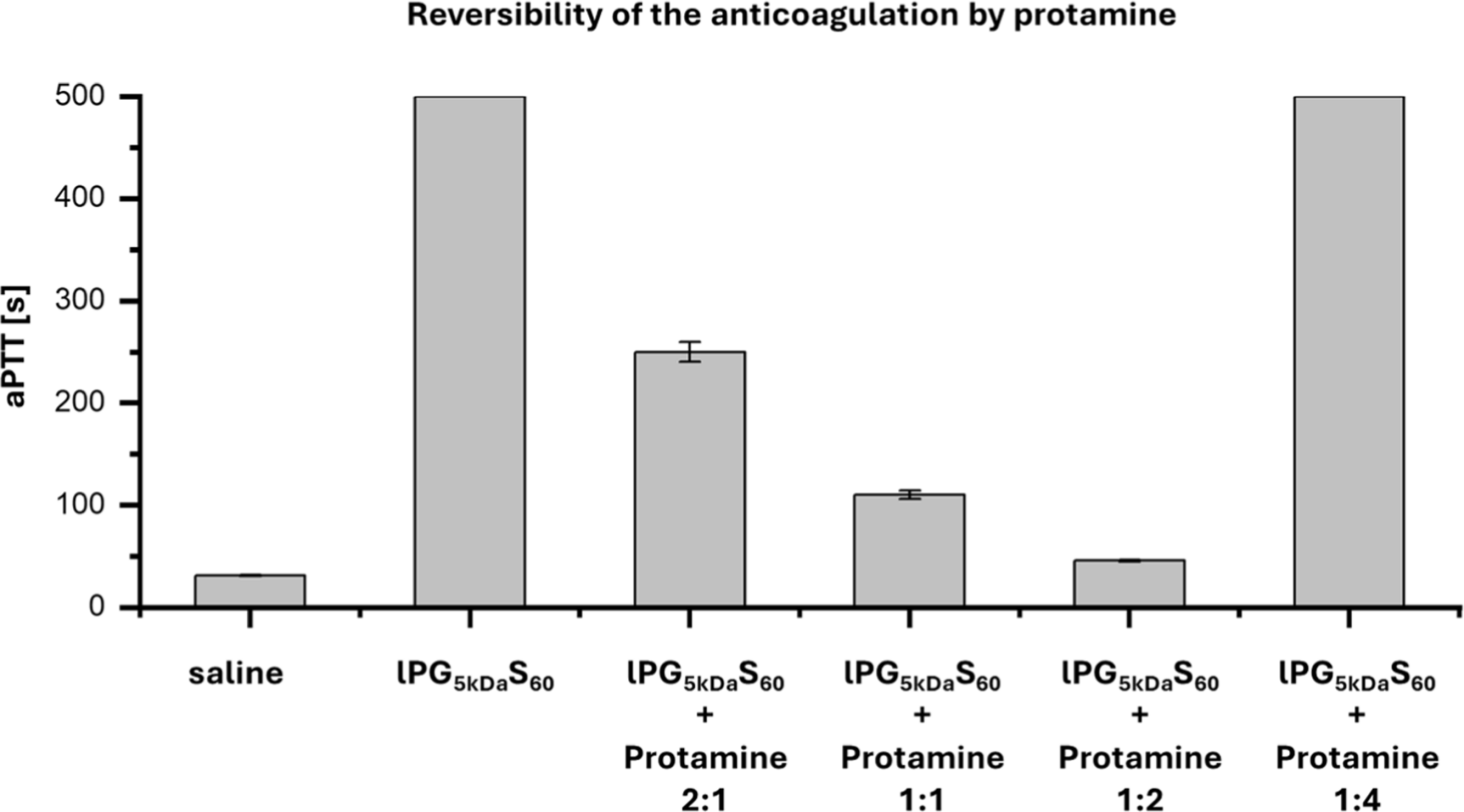
aPTT of PPP in the presence of saline control, lPG_5 kDa_S_60_ (50 μg/mL), and varying molar ratios of lPG_5 kDa_S_60_ (50 μg/mL) to protamine (2:1, 1:1, 1:2, 1:4). Measurements were terminated after 500 s. All experiments were performed as biological triplicates (*n* = 3 (with three technical replicates)). All data represent mean ± standard deviation.

**Table 4:** aPTT of lPGS and Protamine in PPP at Varying Ratios

sample	ratio	c (anticoagulant) [mg/mL]	aPTT [s]
PBS control		0.05	31.2 ± 0.6
lPGS_5 kDa_S_60_		0.05	>500
lPGS_5 kDa_S_60_ + protamine	2:1	0.05	250 ± 10
lPGS_5 kDa_S_60_ + protamine	1:1	0.05	110 ± 4
lPGS_5 kDa_S_60_ + protamine	1:2	0.05	45.6 ± 1.1
lPGS_5 kDa_S_60_ + protamine	1:4	0.05	>500

The observed reduction in the amount of protamine needed to neutralize lPGS_5 kDa_S_60_ compared to UFH is potentially advantageous since protamine application can induce life-threatening side effects such as systemic hypertension, catastrophic pulmonary vasoconstriction, or allergic reactions.^45–47^ Reducing the applied amount of protamine would most likely also reduce the frequency and severity of adverse effects.

Additional SPR experiments investigating the interaction with immobilized protamine confirmed the increased affinity for lPGS_5 kDa_S_60_ (K_D_ = 0.56 nM) over UFH (K_D_ = 3.6 nM) (Figure S16). The increased activity of protamine for the reversal of the anticoagulant effect of lPGS_5 kDa_S_60_ likely arises from its higher density of negative charges compared to UFH, leading to stronger electrostatic interactions.

## Conclusions

We report lPGS as an effective anticoagulant alternative to heparins with similar potency and without the batch variability or disease risks inherent to animal-derived compounds. The detailed structure-to-activity analysis of branched and linear PGS unveiled that the anticoagulant effect strongly increases with the flexibility of the polymer as well as the molecular weight up to a core PG size of 10 kDa. The anticoagulant activity was first determined in vitro using aPTT in platelet poor plasma and then in whole blood by TEG. The increasing value of aPTT based on increased flexibility and increased chain length initially led to the conclusion of a mechanism of action similar to that of the bridging mechanism of heparin with both ATIII and FIIa. However, following chromogenic assays investigating the activity of FIIa, FXa, and ATIII in the presence of PGS revealed their ATIII-independent activity and FIIa selective nature. This mechanism of action was also confirmed by SPR binding studies, where poly­(glycerol)­sulfates were found to bind solely to FIIa.

Besides the general benefits of batch-continuity of a synthetic molecule over the animal-derived UFH, a further potential advantage over the application of heparins is the ability of tunable pharmacokinetics by the inclusion of biodegradable sections to the PG backbone, for example, by introduction of cleavable caprolactone moieties.^48,49^

Finally, the ability of FDA-approved protamine to reverse the anticoagulant activity of lPGS fulfills another requirement of heparin alternatives in surgery. The reduced amount of necessary protamine for anticoagulant reversal would also present an advantage due to the often-experienced unwanted side effects following protamine injection. Furthermore, the single azide end group could easily be further functionalized for coating applications analogous to the heparin coating of coronary stents to reduce the rate of restenosis or thrombosis.

## Supplementary Material



## Notes

Polymer synthesis and characterization, SPR experiments, TEG and aPTT experiments, and cell viability assays (PDF)
