# Supplementary material for: The Structure–Activity Relationship and Anticoagulation Mechanism of Polyglycerol Sulfates of Different Architectures
Source: Biomacromolecules. 2026 Apr 29;27(5):3367–75. doi: 10.1021/acs.biomac.6c00308 (PMC13169359; doi:10.1021/acs.biomac.6c00308)
Supplement: Supplementary file 1 [file bm6c00308_si_001.pdf]

Supporting Information for

Structure activity relationship and anticoagulation mechanism of polyglycerol sulfates of different architectures

[†]Clemens Krage, [†§]Marie Weinhart, [‡]Benjamin F.L.Lai, [†]Anja Stöshel, [†]Katharina Achazi, [‡,#]Jayachandran N. Kizhakkedathu*, [†]Rainer Haag*

† Institute für Chemie und Biochemie, Freie Universität Berlin, Takustrasse 3, D-14195 Berlin, Germany

‡ Centre for Blood Research, Life Sciences Institute, Department of Pathology and Laboratory Medicine, University of British Columbia, 2350 Health Sciences Mall, Life Sciences Centre, Vancouver, BC V6T 1Z3, Canada

The School of Biomedical Engineering, University of British Columbia, 2350 Health Sciences Mall, Life Sciences Centre, Vancouver, BC V6T 1Z3, Canada

§ Leibniz Universität Hannover, Institute of Physical Chemistry and Electrochemistry, Hannover 30167, Germany

Corresponding authors: Jayachandran E. Kizhakkedathu, Rainer Haag

Synthesis

Linear polyglycerol sulfate

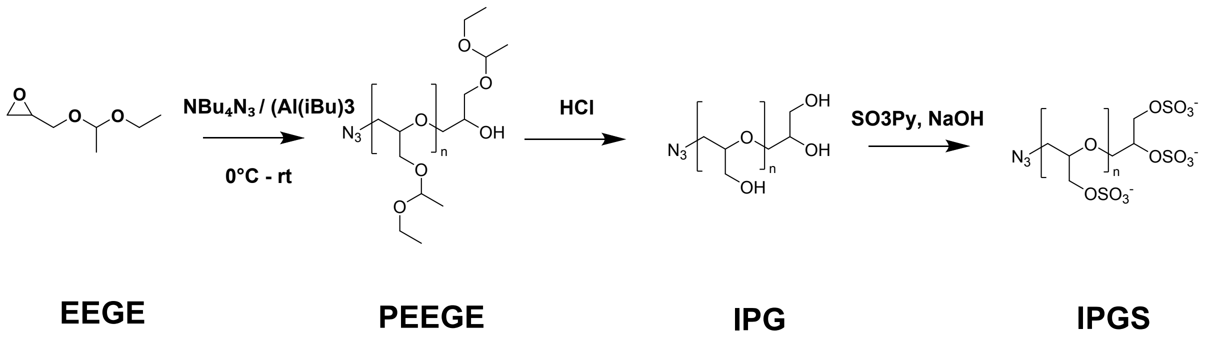

SI scheme 1: synthesis of linear polyglycerol sulfate

The synthesis of linear polyglycerol sulfate has been performed as described in previous publications[1, 2]:

Polymerization:
Dry tetrabutylammonium azide (NBu_4N_3) (1.1 g, 1 equiv.) was dissolved in anhydrous toluene by sonication and subsequently cooled to 0 °C. Ethoxyethylglycidyl ether (EEGE) (7.92 mg, 14 equiv.) was slowly added to the solution under vigorous stirring, followed by rapid addition of triisobutylaluminum (17.6 mL, 5 equiv.). After stirring for 16 h, water (3 mL, 5 equiv.) was added to terminate the reaction, and the resulting precipitate was removed by filtration. The solvent was removed under reduced pressure. The crude product, poly(ethoxyethylglycidyl ether) (PEEGE), was purified by dialysis, followed by lyophilization. ^1H NMR (400 MHz, $CDCl_3$, δ): 1.14–1.17 (t, 3H), 1.25–1.26 (d, 3H), 3.40–3.65 (m, 7H), 4.66 (d, 1H).

Deprotection
PEEGE (1.2 g, 1 equiv.) was dissolved in ethanol (12 mL), followed by the addition of water (4.8 mL) and 37% aqueous hydrochloric acid (1.4 mL). The mixture was stirred overnight in an open flask. The solvent was removed under reduced pressure, and the residue was dissolved in water and dialyzed against water for 48 h. Lyophilization afforded linear polyglycerol (lPG). ^1H NMR (400 MHz, D_2O, δ): 3.56–3.67 (m, 5H).

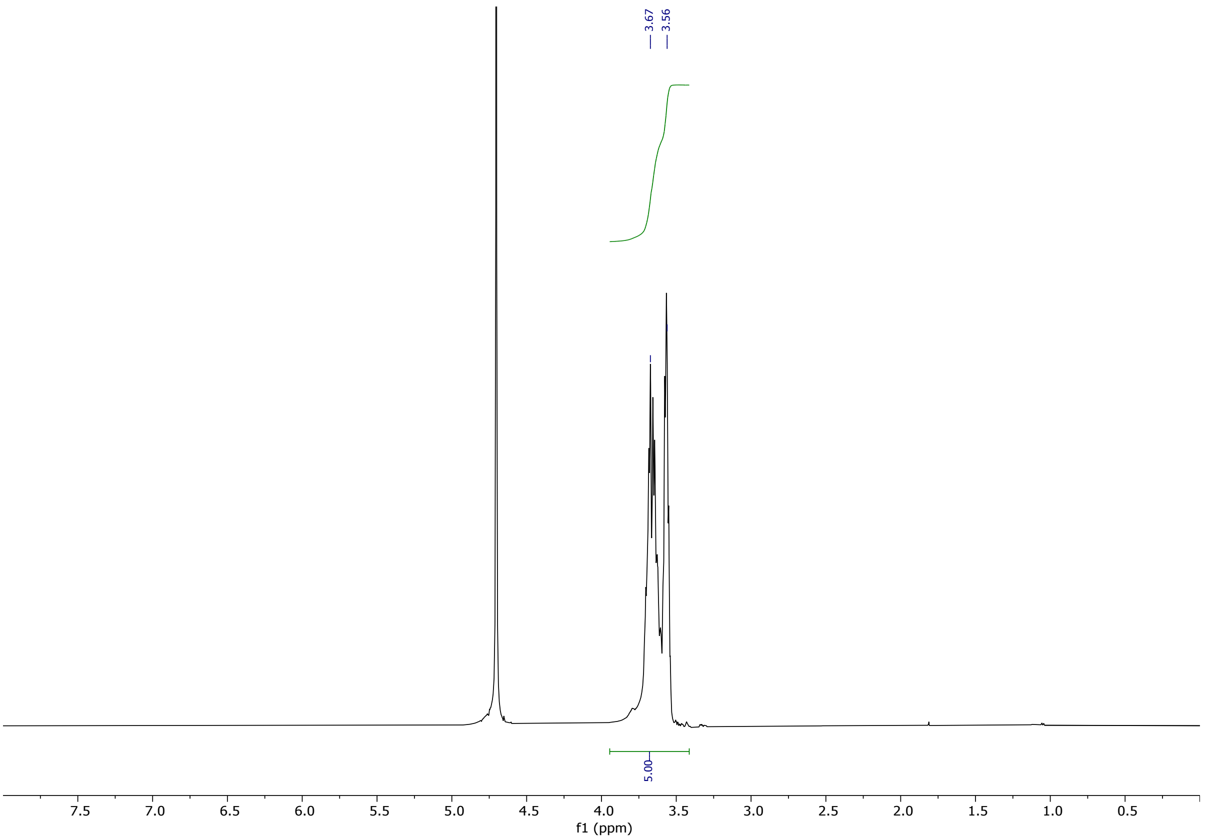

SI Figure 1: ^{1}H NMR (500 MHz, D2O, δ (ppm)) of lPG$_{5kDa}$

Sulfation

lPG (1 g, 1 eq.) was dissolved in anhydrous DMF and heated to 60°C under stirring. SO_3–pyridine complex was added dropwise (2.15 g, 1.5 equiv.), and the reaction mixture was allowed to warm to room temperature and stirred over night. The reaction was terminated by the addition of water and pH adjustion to pH = 8 by 1 M sodium hydroxide. The aqueous phase was purified by dialysis against brine followed by deionized water for 48 h, yielding the sulfated polymer (lPGS) after lyophilization. ^{1}H NMR (400 MHz, D_2O, δ): 4.12 – 4.25 (m, 2H), 3.78 – 3.89 (m, 3H).

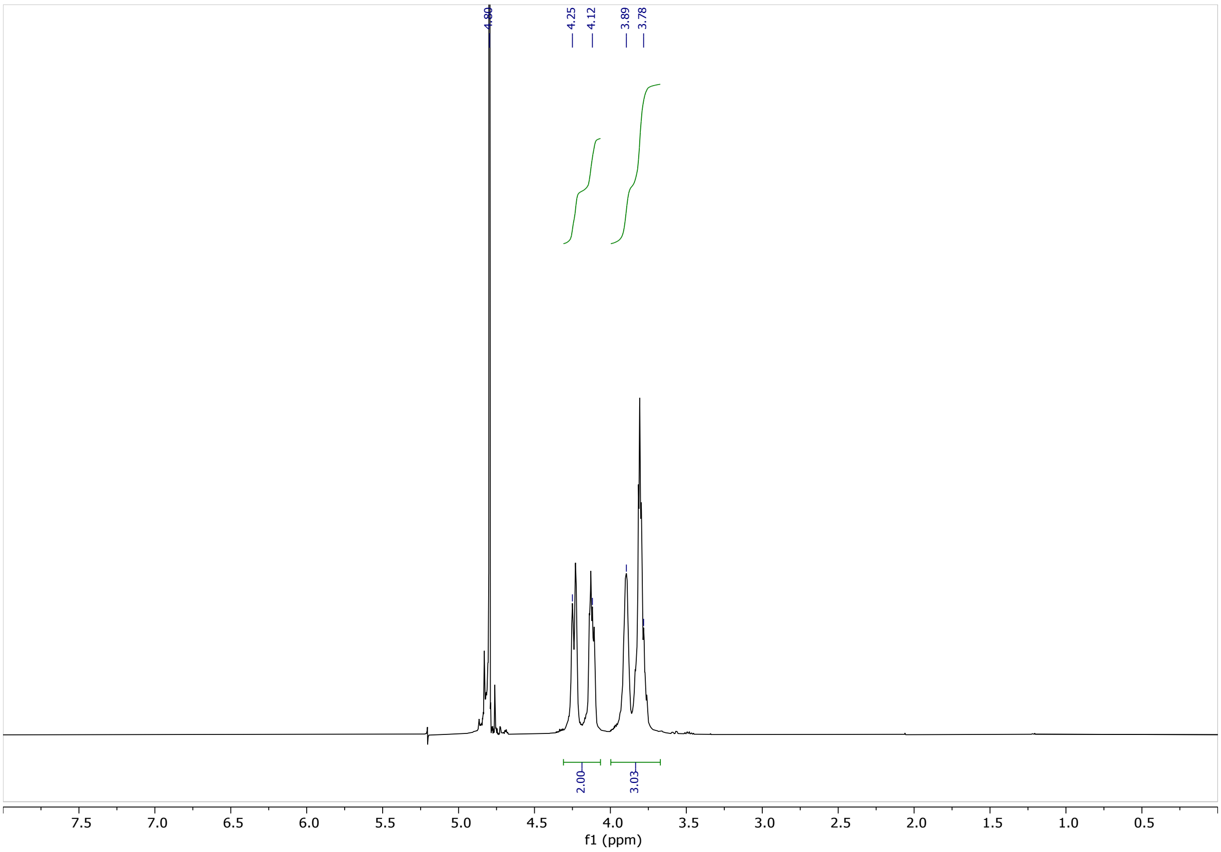

SI Figure 2: ^{1}H NMR (500 MHz, D2O, δ (ppm)) of lPG$_{5kDa}$S$_{60}$

Dendritic polyglycerol sulfate

Dendritic PG of the average molecular weight of 5 kDa was prepared as previously reported from our group. [3-5] The sulfation was performed as described above for lPGS. ^{1}H NMR (500 MHz, D2O, δ (ppm)): 3.70-4.71 (m, 5H).

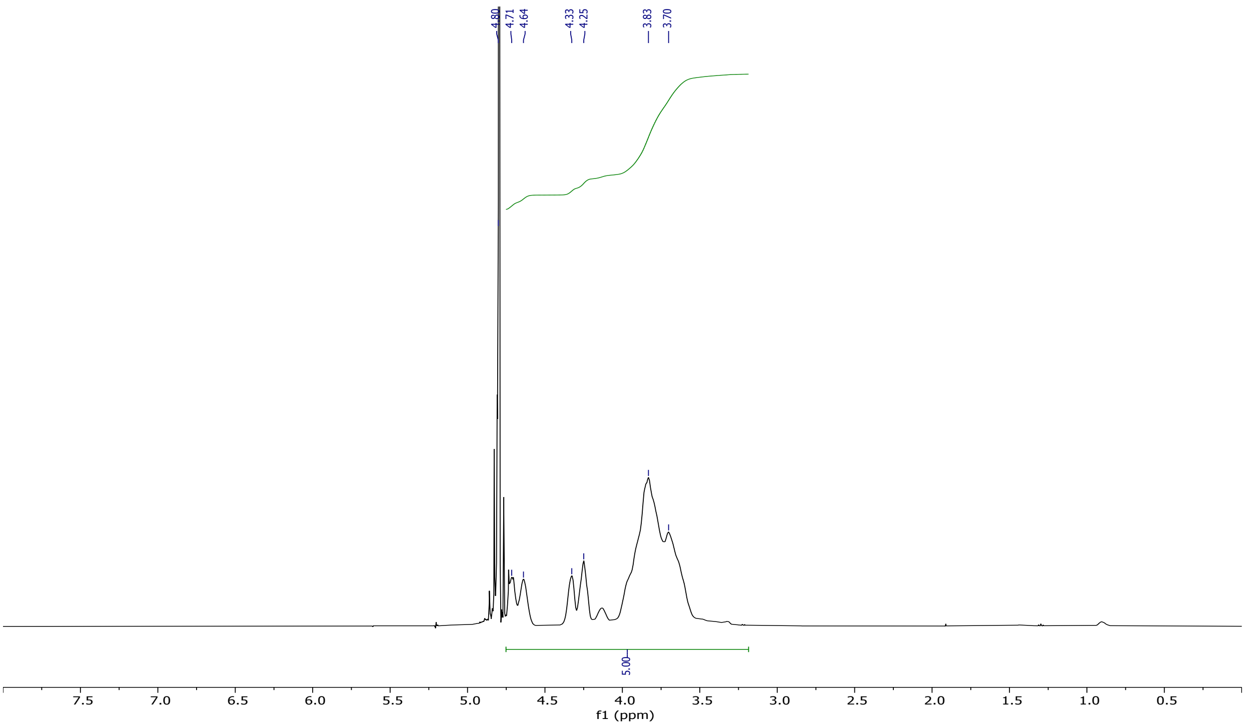

SI Figure 3: ^{1}H NMR (500 MHz, D2O, δ (ppm)) of dPG$_{5kDa}$S$_{60}$

Four-star polyglycerol sulfate

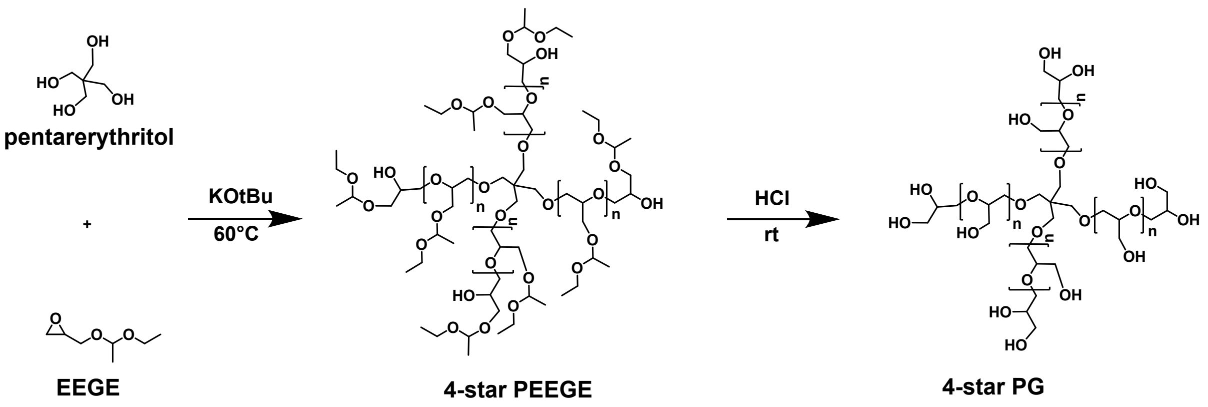

SI Scheme 2: Synthesis of 4-star PG

Polymerization

Dry pentaryhtritol (0.135 g, 1 equiv.) was dissolved in anhydrous THF and subsequently heated to 60 °C. Potassium t-butoxide (KOtBu) (40 mL, 0.4 equiv.) was slowly added to the solution under vigorous stirring. Ethoxyethylglycidyl ether (EEGE) (9.68 g, 68 equiv.) was then rapidly added. After stirring for 96 h, Methanol (3 mL, 5 equiv.) was added to terminate the reaction. The solvent was removed under reduced pressure. The crude product, 4-star PEEGE, was purified by dialysis, followed by lyophilization. ^{1}H NMR (400 MHz, $CDCl_3$, δ): 1.17–1.30 (m, 6H), 3.43–3.48 (m, 2H), 3.54-3.67 (m, 5H), 4.67-4.70 (q, 1H).

The following deprotection and sulfation were performed as described for linear polyglycerol sulfate.

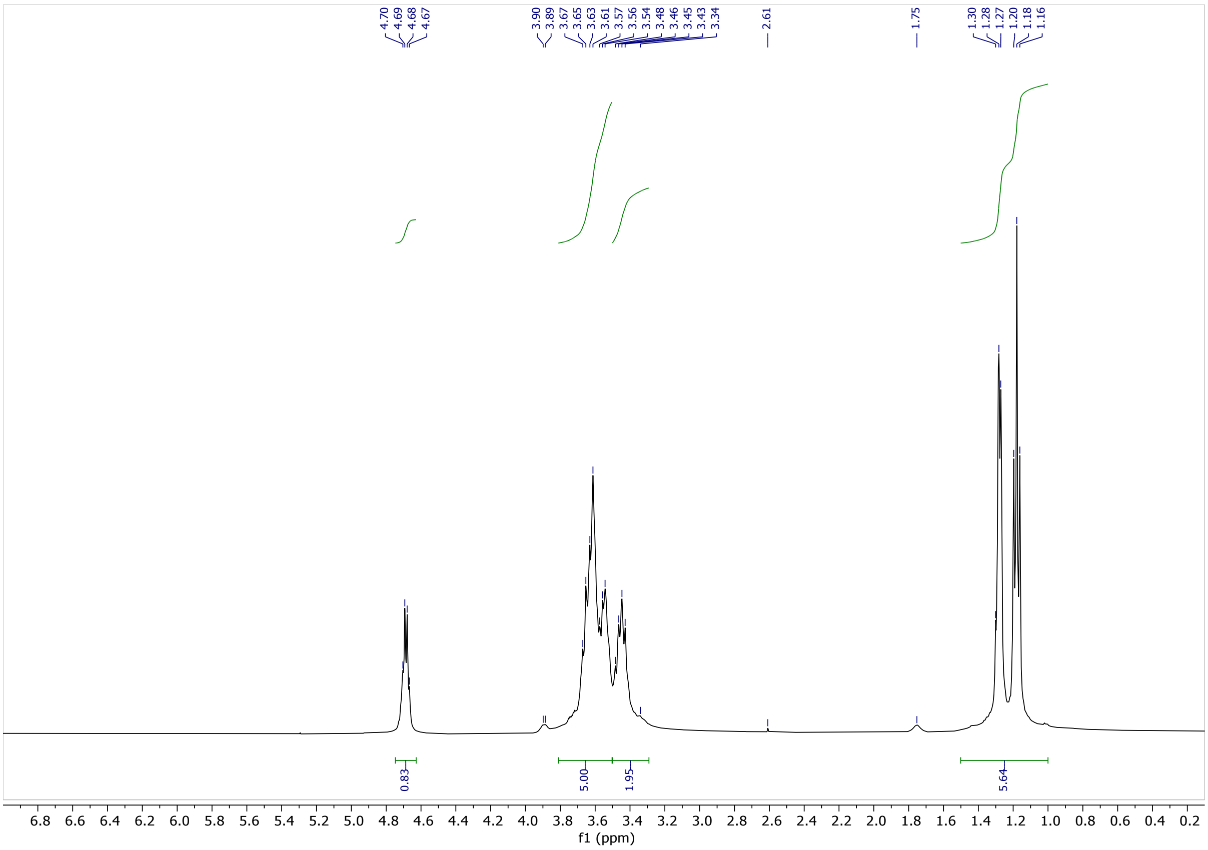

SI Figure 4: ^{1}H NMR (500 MHz, D2O, δ (ppm)) of protected four-star polyglycerol (4-star PEEGE)

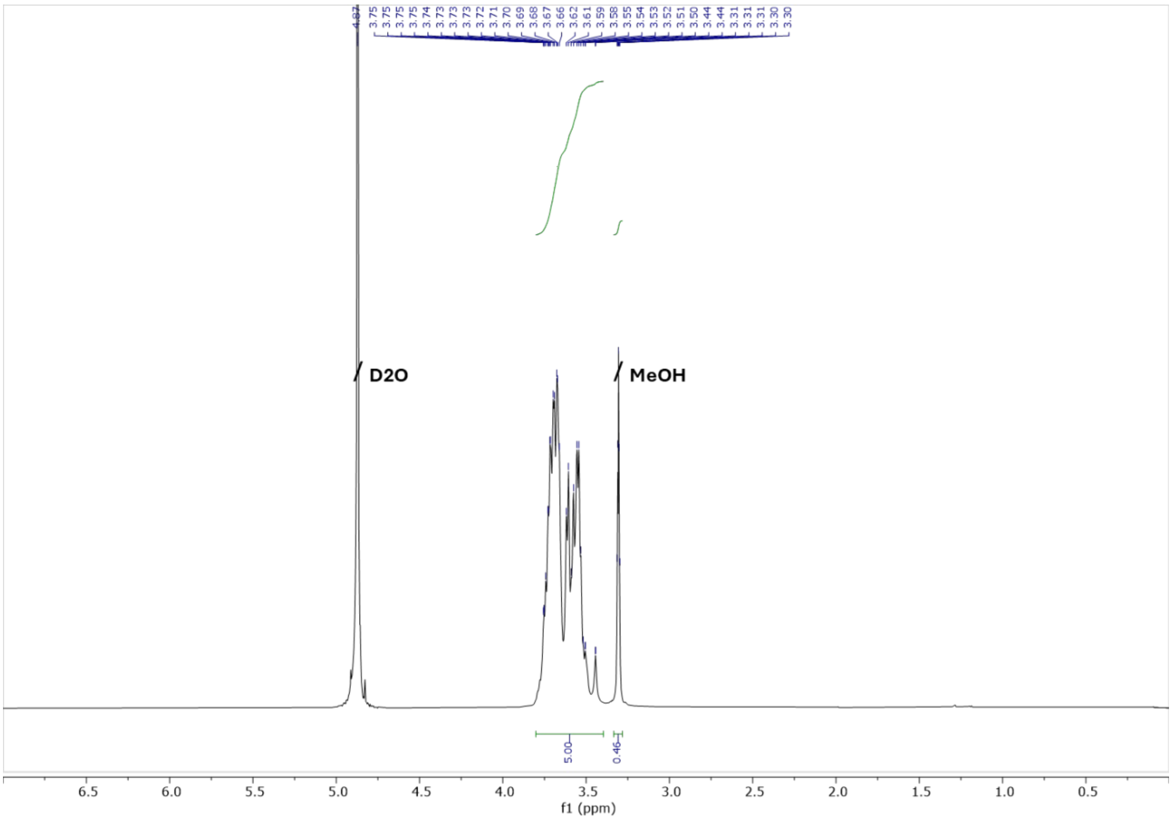

SI Figure 5: ^{1}H NMR (500 MHz, D2O, δ (ppm)) of deprotected four-star polyglycerol

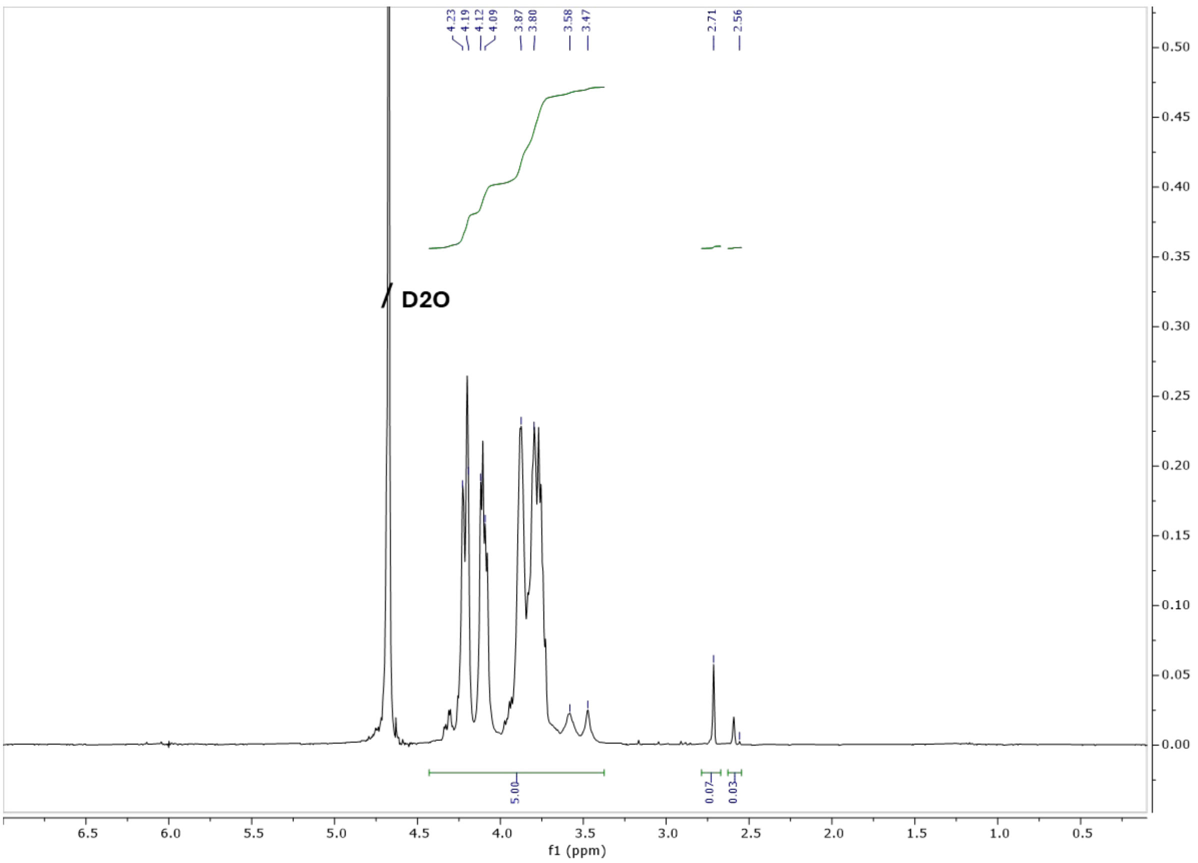

SI Figure 6: ^{1}H NMR (500 MHz, D2O, δ (ppm)) of four-star polyglycerol sulfate (4-star $PG_{12kDa}S_{67}$)

Six-star polyglycerol sulfate

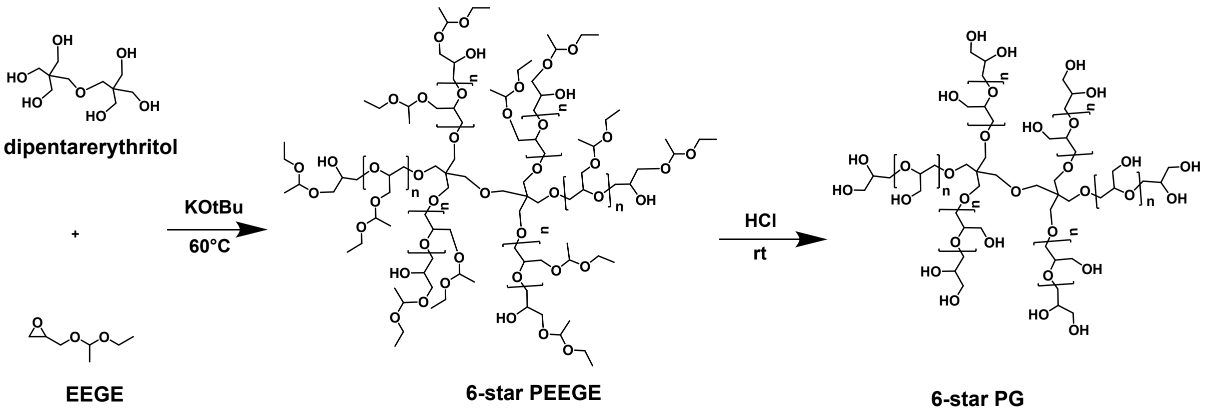

SI Scheme 3: Synthesis of 6-star PG

Polymerization

Dry Dipentaerythritol (0.236 g, 1 equiv.) was dissolved in anhydrous THF and subsequently heated to 60 °C. Potassium t-butoxide (KOtBu) (40 mL, 0.4 equiv.) was slowly added to the solution under vigorous stirring. Ethoxyethylglycidyl ether (EEGE) (9.77 g, 72 equiv.) was then rapidly added. After stirring for 96 h, water (3 mL, 5 equiv.) was added to terminate the reaction. The solvent was removed under reduced pressure. The crude product, 6-star PEEGE,

was purified by dialysis, followed by lyophilization. ^{1}H NMR (400 MHz, $CDCl_3$, δ): 1.17–1.67 (m, 6H), 3.32-3.49 (m, 2H), 3.55–3.90 (m, 5H), 4.67-4.73 (q, 1H).

The following deprotection and sulfation were performed as described for linear polyglycerol sulfate.

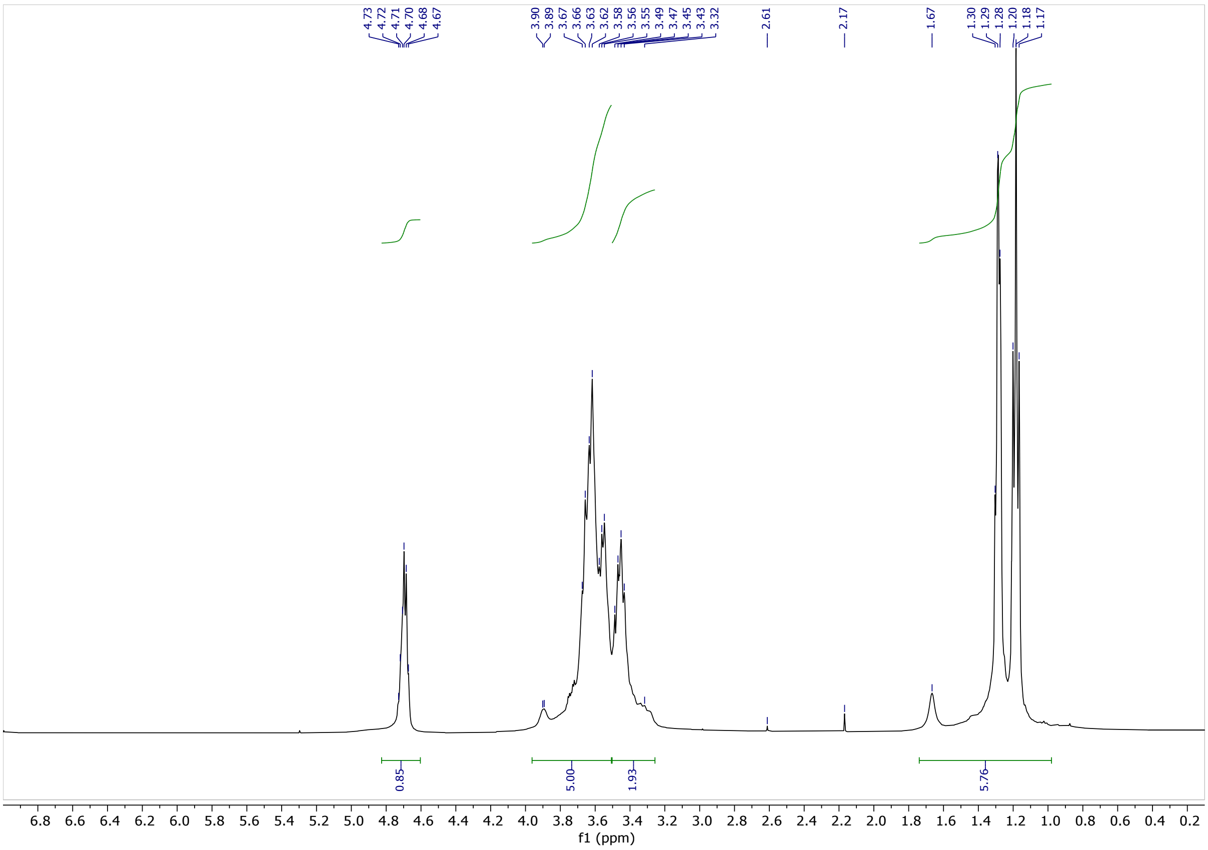

SI Figure 7: ^{1}H NMR (500 MHz, D2O, δ (ppm)) of protected six-star polyglycerol (six-star PEEGE).

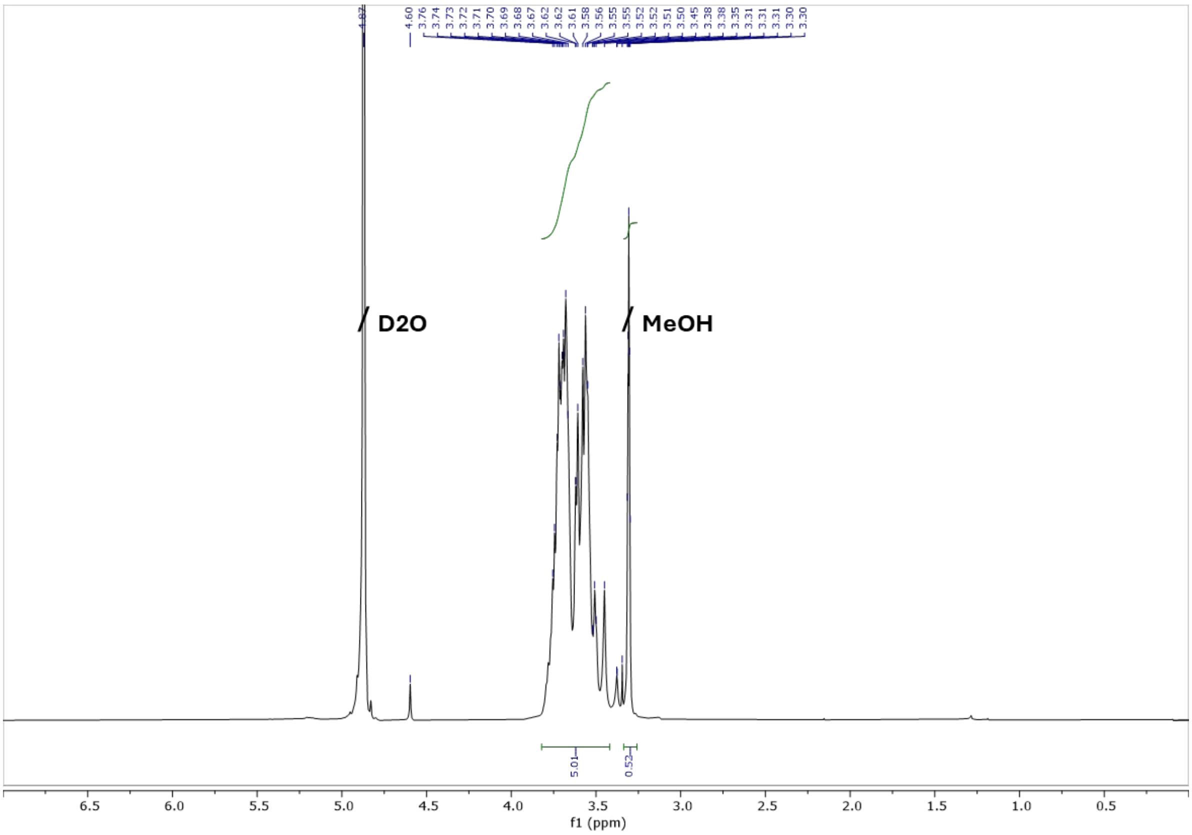

SI Figure 8: ^{1}H NMR (500 MHz, D2O, δ (ppm)) of deprotected six-star polyglycerol.

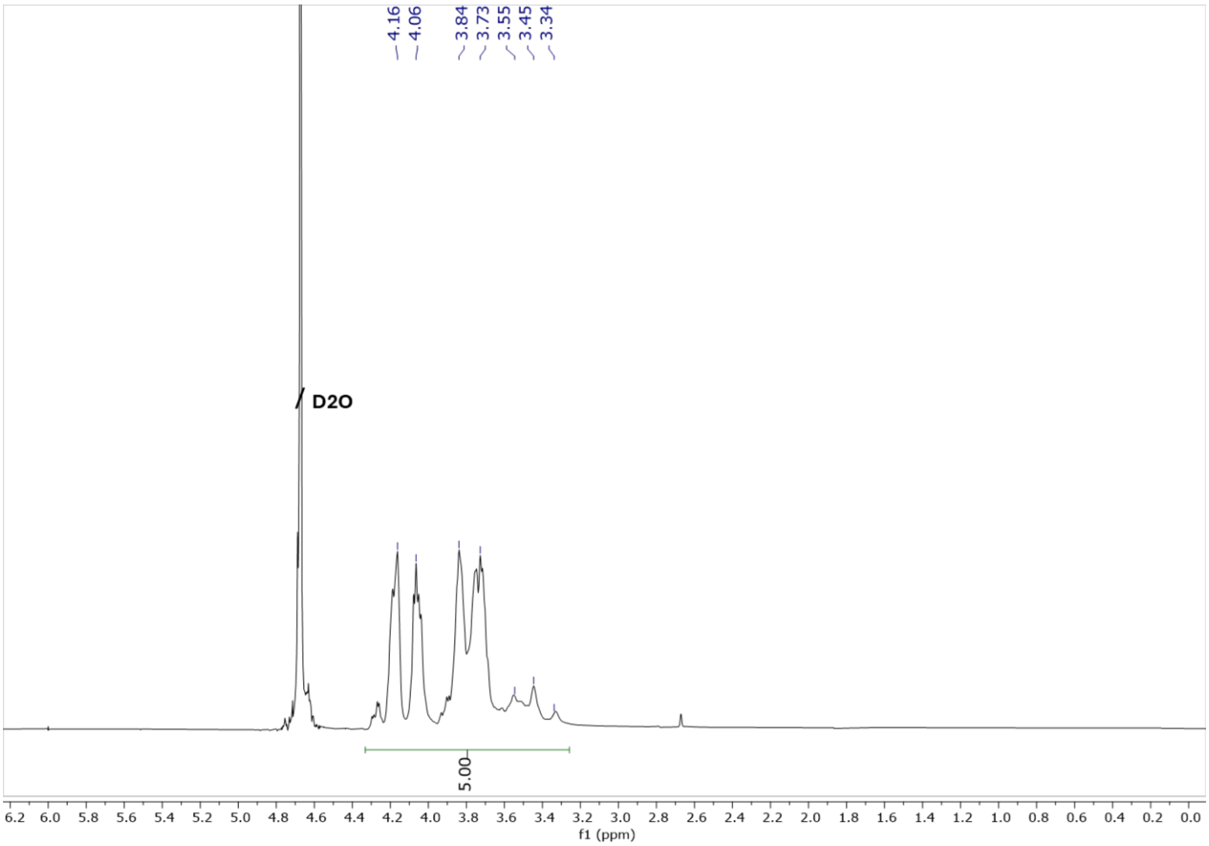

SI Figure 9: ^{1}H NMR (500 MHz, D2O, δ (ppm)) of sulfated six-star polyglycerol sulfate (6-star $PG_{12kDa}S_{67}$).

GPC

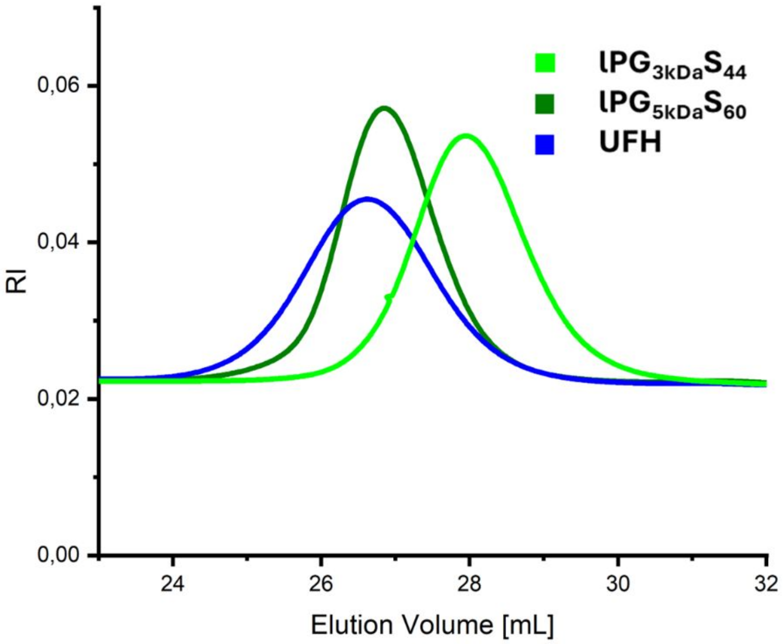

SI Figure 10: Representative gel permeation chromatography results for UFH (blue), $lPG_{3kDa}S_{44}$ (bright green) and $lPG_{5kDa}S_{60}$ (dark green). Pullulan was used as a reference standard. Water was used as solvent.

Thromboelastography

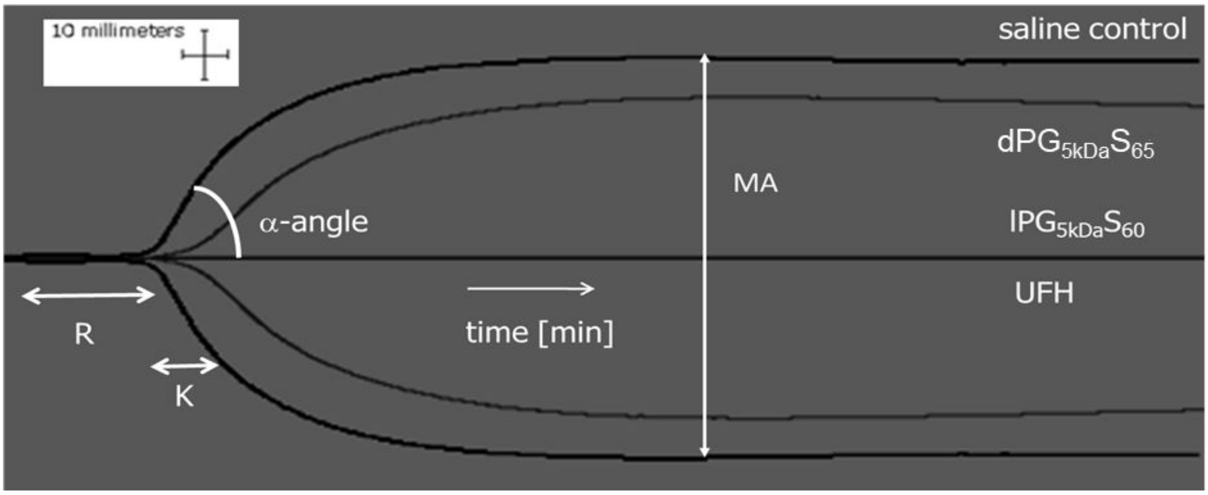

SI Figure 11: Representative Thromboelastogram of whole blood in the presence of UFH (2 IU/mL), $lPG_{5kDa}S_{60}$ (0.05 mg/mL) and $dPG_{5kDa}S_{65}$ (0.05 mg/mL) and the nontreated saline control shows a comparable anticoagulant effect of UFH and $lPG_{5kDa}S_{60}$. While UFH and $lPG_{5kDa}S_{60}$ completely inhibit clot formation, while $dPG5_{kDa}S_{65}$ slightly increases the clot formation time (R-value) compared to the saline control.

Cytotoxicity

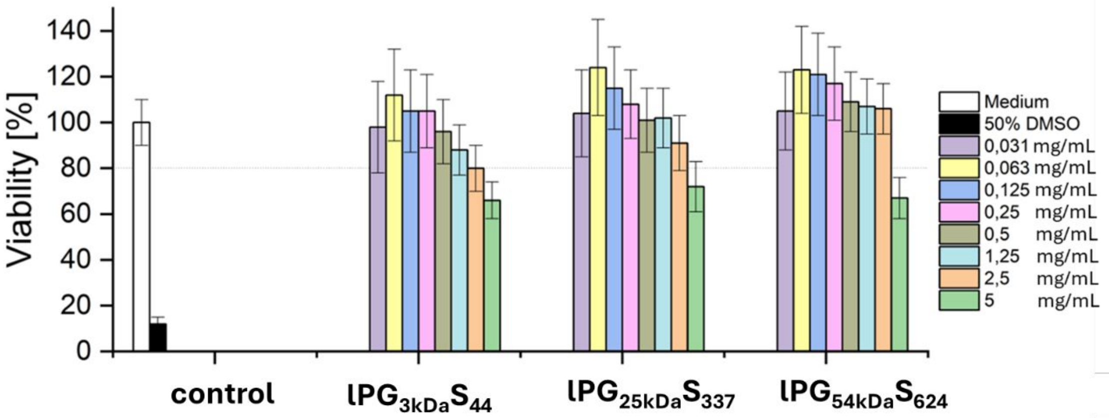

SI Figure 12: Cytotoxicity was investigated for human umbilical vein endothelial cells (HUVEC) (ThermoFisher) via an MTS assay after 48 h incubation time (37°C). Experiments were performed as biological triplicates (n=9), Error bars represent the standard error.

SPR spectroscopy

ATIII binding

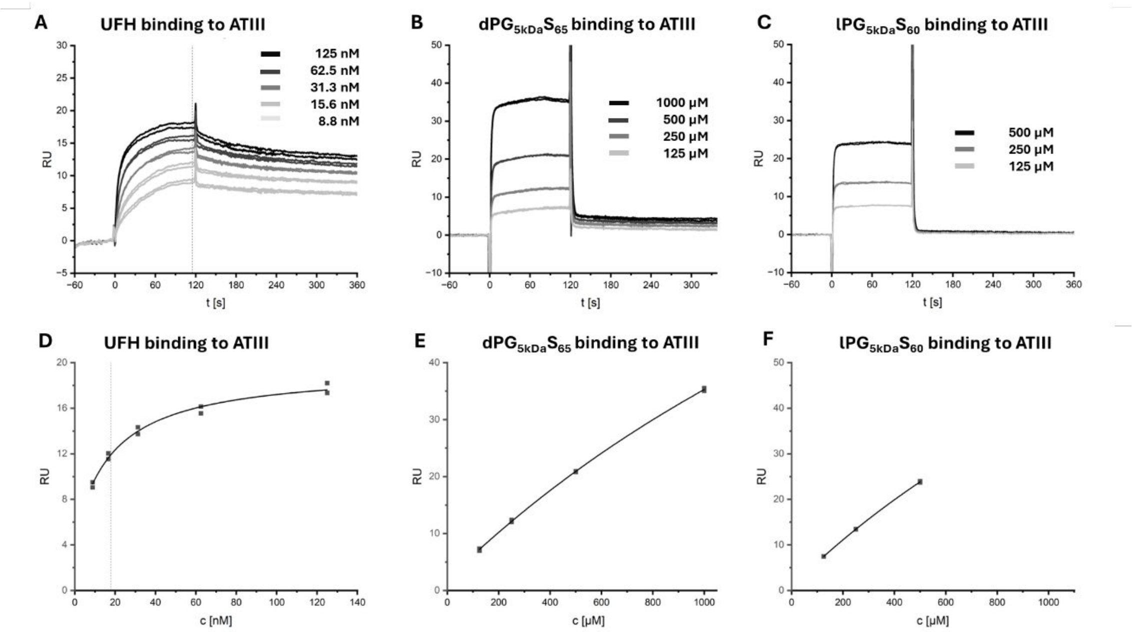

SI Figure 13: SPR sensorgrams (A-C) and Affinity fits (D-F) of the binding of UFH (K_D = 18 nM), lPG$_{5kDa}$S$_{60}$ (K_D > mM) and dPG$_{5kDa}$S$_{65}$ (K_D > mM) to immobilized ATIII. High affinity binding was only observed for UFH, while the affinity fits of lPG$_{5kDa}$S$_{60}$ and dPG$_{5kDa}$S$_{65}$ show no saturation up to 500 µM, indicating a K_D in the millimolar range. Affinity fitting was performed by the BIAevaluation software by plotting the steady state response against the concentration. All experiments were performed as duplicates.

FIIa binding

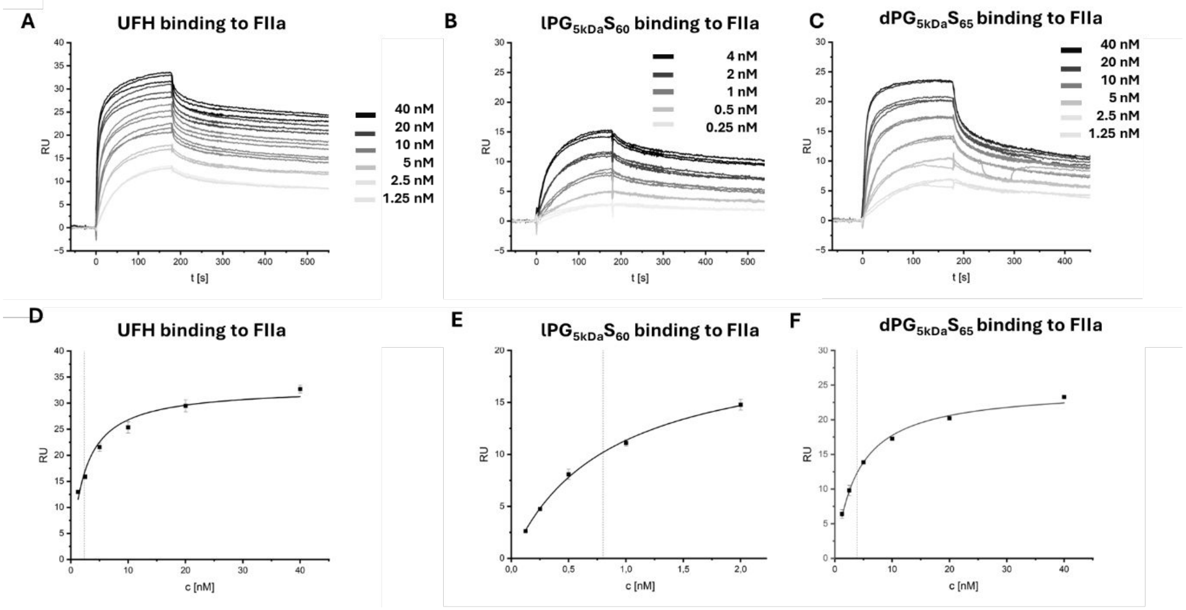

SI Figure 14: SPR sensorgrams (A-C) and Affinity fits (D-F) of the binding of UFH (K_D = 2.3 nM), E) lPG$_{5kDa}$S$_{60}$ (K_D = 0.8 nM) and F) dPG$_{5kDa}$S$_{65}$ (K_D = 3.9 nM). to immobilized FIIa. Affinity fitting was performed by the BIAevaluation software by plotting the steady state response against the concentration. All experiments were performed as triplicates.

Reversability of anticoagulant activity by Protamine (apTT of PPP)

Table 1: aPTT of lPGS and protamine in PPP at varying ratios.

Sample	Ratio	c (anticoagulant) [mg/mL]	aPTT [s]
PBS control		0.05	31.2 ± 0.6
$lPGS_{5kDa}S_{60}$		0.05	> 500
$lPGS_{5kDa}S_{60}$ + Protamine	2:1	0.05	250 ± 10
$lPGS_{5kDa}S_{60}$ + Protamine	1:1	0.05	110 ± 4
$lPGS_{5kDa}S_{60}$ + Protamine	1:2	0.05	45.6 ± 1.1
$lPGS_{5kDa}S_{60}$ + Protamine	1:4	0.05	> 500

Reversability of anticoagulant activity by Protamine (TEG)

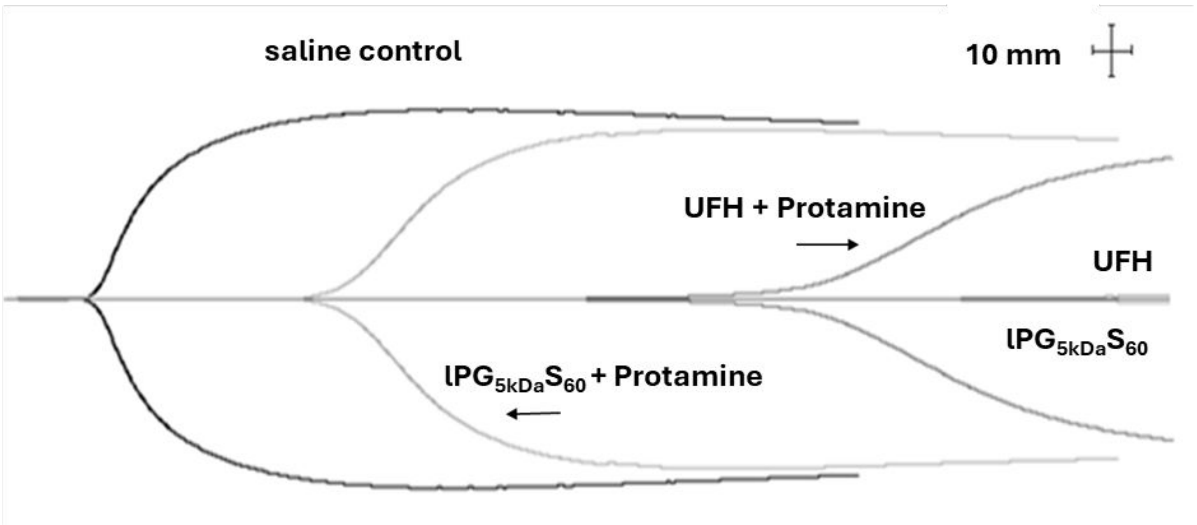

SI Figure 15 Thromboelastogram of whole blood after adding $lPGS_{5kDa}S_{60}$ (0.05 mg/mL), UFH (0.05 mg/mL), $lPGS_{5kDa}S_{60}$ + protamine (0.05 mg/mL, 2.5:1) or UFH + protamine (0.05 mg/mL, 2:1). TEG parameters are presented in Table 1. Only single TEG measurements were performed to confirm the observed effect on the aPTT (Figure 5).

SI Table 1: TEG Parameters in the presence and absence of protamine

Sample	c	R value [min]	K value [min]	MA [mm]	α-angle
PBS control	-	11.3	3	55.6	48.9
$lPGS_{5kDa}S_{60}$	0.05 mg/mL	140.2	36.9	n.a.	n.a.
$lPGS_{5kDa}S_{60}$ + Protamine 2.5:1	0.05 mg/mL	37.9	8.2	49.7	27.4
UFH	0.05 mg/mL	191.1	n.a.	n.a.	n.a.
UFH + Protamine (2:1)	0.05 mg/mL	87.7	18.8	42.9	9.5

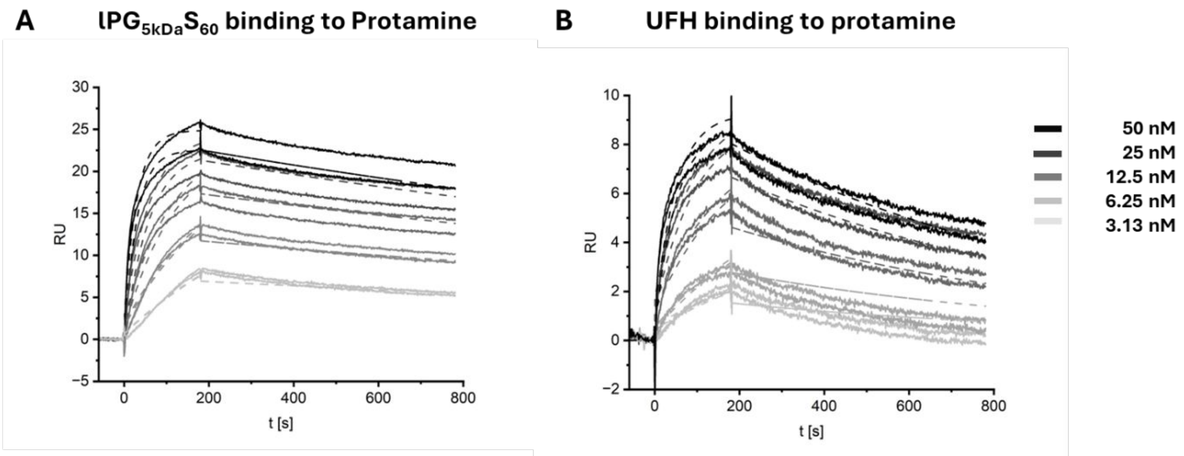

SI Figure 16: SPR Sensorgrams of binding of immobilized protamine to A) lPGS (K_D = 0.56 nM, k_a = 6.7x10^5 (1/Ms), k_d = 3.8x10^{-4} (1/s)) and B) UFH (K_D = 3.6 nM, k_a = 2.9x10^5 (1/Ms), k_d = 1.0x10^{-3} (1/s). Kinetic constants k_a, k_d and equilibrium dissociation constants were determined by a 1:1 global kinetic fit (dashed lines) by the BIAevaluation software. All experiments were performed as duplicates.

References

(1) Pouyan, P.; Zemella, A.; Schloßhauer, J. L.; Walter, R. M.; Haag, R.; Kubick, S. One to one comparison of cell-free synthesized erythropoietin conjugates modified with linear polyglycerol and polyethylene glycol. *Sci Rep-Uk* **2023**, *13* (1), 6394. DOI: 10.1038/s41598-023-33463-x.

(2) Nie, C.; Pouyan, P.; Lauster, D.; Trimpert, J.; Kerkhoff, Y.; Szekeres, G. P.; Wallert, M.; Block, S.; Sahoo, A. K.; Dernedde, J.; et al. Polysulfates Block SARS-CoV-2 Uptake through Electrostatic Interactions. *Angewandte Chemie International Edition* **2021**, *60* (29), 15870-15878. DOI: https://doi.org/10.1002/anie.202102717.

(3) Haag, R.; Sunder, A.; Stumbé, J. F. An approach to glycerol dendrimers and pseudo-dendritic polyglycerols. *J Am Chem Soc* **2000**, *122* (12), 2954-2955. DOI: DOI 10.1021/ja994363e.

(4) Sunder, A.; Hanselmann, R.; Frey, H.; Mülhaupt, R. Controlled synthesis of hyperbranched polyglycerols by ring-opening multibranching polymerization. *Macromolecules* **1999**, *32* (13), 4240-4246. DOI: DOI 10.1021/ma990090w.

(5) Wallert, M.; Plaschke, J.; Dimde, M.; Ahmadi, V.; Block, S.; Haag, R. Automated Solvent-Free Polymerization of Hyperbranched Polyglycerol with Tailored Molecular Weight by Online Torque Detection. *Macromol Mater Eng* **2021**, *306* (7). DOI: ARTN 2000688 10.1002/mame.202000688.
